# Annotations

**Published:** 1886-12-11

**Authors:** 


### Annotations.

**Coventry's Care for its Sick Children.**

The bazaar in aid of the Coventry Children's Hospital, which Mrs. Gulson has promoted, has resulted, we understand, in the receipt of all the money required for the Endowment Fund, with the exception of £200, which Alderman Gulson has generously made up. Such a result is satisfactory and encouraging, and we venture to congratulate not only Mrs. Gulson, but the people of Coventry, who now enjoy the blessings of good trade and abundant employment, that they have made it a matter of conscience to raise the endowment fund to the required amount, as explained in these columns on the 13th ult. This is one more proof that good deeds, when performed with spirit and sound judgment, will commend themselves to the people of England, and ensure to those who do them an adequate public recognition and support. Mrs. Gulson certainly deserves some special recognition of the successful accomplishment of her life's work, and we are glad to find that Coventry has shown such an excellent public spirit as it has done in this matter.

**The First Medical General Election.**

All that concerns the welfare of the medical profession must prove of interest to the readers of this Journal. It is satisfactory, therefore, to note, that of the 18,000 medical practitioners on the register in England, some 70 per cent., or upwards of 13,000, have taken part in the general election, under the new Medical Act, to return three representatives to sit on the General Council of Medical Education and Registration. The successful candidates were,—for England: Mr. C. G. Wheelhouse of Leeds, 8,548 votes; Sir B. W. Foster of Birmingham, 7,718 votes; and Dr. T. G. Glover of Highbury, 6,614 votes; for Scotland: Dr. Bruce, 642 votes; and, for Ireland: Dr. Kidd, 911 votes. We heartily congratulate these gentlemen upon their election, and especially Dr. Glover, whose knowledge of the work of the Medical Council and the Medical Acts is probably unsurpassed. Dr. Glover is, besides, in the highest and best sense of the term, a worthy representative of the whole body of general practitioners throughout the country. Let us hope that the new leaven may infuse new life and energy into the Medical Council, and thus prove how useful this body can be made when it is aroused to a sense of duty and public spirit, for the good of the whole profession and the public at large.

---

Dr. Donovan, despite his poverty, was a bit of a wag. "Your family," said a friend, "is Irish, and, I believe, illustrious. How is it then, that you do not follow the custom of your country, and style yourself Dr. O'Donovan?" "Really, my good sir, I am so deeply in debt," he replied, "that I do not care to *owe* any more."

